# Age-Related Declines in Lower Limb Muscle Function are Similar in Power and Endurance Athletes of Both Sexes: A Longitudinal Study of Master Athletes

**DOI:** 10.1007/s00223-021-00907-3

**Published:** 2021-09-09

**Authors:** Alex Ireland, Uwe Mittag, Hans Degens, Dieter Felsenberg, Ari Heinonen, Erika Koltai, Marko T. Korhonen, Jamie S. McPhee, Igor Mekjavic, Rado Pisot, Rainer Rawer, Zsolt Radak, Bostjan Simunic, Harri Suominen, Jörn Rittweger

**Affiliations:** 1 Department of Life Sciences, Musculoskeletal Science and Sports Medicine Research Centre, Manchester Metropolitan University, John Dalton Building, Chester Street, Manchester, M1 5GD UK; 2 Institute of Aerospace Medicine, German Aerospace Center (DLR), Cologne, Germany; 3 Lithuanian Sports University, Kaunas, Lithuania; 4 University of Medicine and Pharmacy of Târgu Mureș, Târgu Mureș, Rumania; 5 Private Praxis ´Osteology and Orphane Bone Diseases´ and Charité – Campus Benjamin Franklin, Centre of Muscle and Bone Research, Humboldt-University Berlin and Free University, Berlin, Germany; 6 Faculty of Sport and Health Sciences, University of Jyväskylä, Jyväskylä, Finland; 7 Research Institute of Sport Science, University of Physical Education, Budapest, Hungary; 8 Gerontology Research Center, Faculty of Sport and Health Sciences, University of Jyväskylä, Jyväskylä, Finland; 9 Department of Sport and Exercise Sciences, Musculoskeletal Science and Sports Medicine Research Centre, Manchester Metropolitan University, Manchester, UK; 10 Department of Automation, Biocybernetics and Robotics, Jozef Stefan Institute, Ljubljana, Slovenia; 11 Department of Biomedical Physiology and Kinesiology, Simon Fraser University, Burnaby, BC Canada; 12 Science and Research Centre Koper, Institute for Kinesiology Research, Koper, Slovenia; 13 Novotec Medical GmbH, Pforzheim, Germany; 14 Department of Pediatrics and Adolescent Medicine, University of Cologne, Cologne, Germany

**Keywords:** Exercise, Ageing, Physical activity, Mobility

## Abstract

The age-related decline in muscle function, particularly muscle power, is associated with increased risk of important clinical outcomes. Physical activity is an important determinant of muscle function, and different types of physical activity *e.g.* power-based versus endurance-based exercise appear to have differential effects on muscle power. Cross-sectional studies suggest that participation in power-based exercise is associated with greater muscle power across adulthood but this has not been investigated longitudinally. We recruited eighty-nine male and female power and endurance master athletes (sprint and distance runners respectively, baseline age 35–90y). Using jumping mechanography, we measured lower limb muscle function during a vertical jump including at least two testing sessions longitudinally over 4.5 ± 2.4y. We examined effects of time, discipline (power/endurance) and sex in addition to two- and three-way interactions using linear mixed-effects models. Peak relative power, relative force and jump height, but not Esslingen Fitness Index (indicating peak power relative to sex and age-matched reference data) declined with time. Peak power, force, height and EFI were greater in power than endurance athletes. There were no sex, discipline or sex*discipline interactions with time for any variable, suggesting that changes were similar over time for athletes of both sexes and disciplines. Advantages in lower limb muscle function in power athletes were maintained with time, in line with previous cross-sectional studies. These results suggest that improvements in lower limb function in less active older individuals following power-based training persist with continued adherence, although this requires further investigation in interventional studies.

**Supplementary Information:**

The online version contains supplementary material available at 10.1007/s00223-021-00907-3.

## Introduction

With increasing age the prevalence of low muscle mass, quality and function, known clinically as sarcopenia, increases dramatically and is associated with increased risk of multiple important clinical outcomes including functional decline, falls, hospitalisation and premature mortality [1]. Given ageing populations worldwide, sarcopenia represents a substantial and growing burden to individuals and healthcare systems. In the United Kingdom alone, annual direct healthcare costs associated with muscle weakness are estimated to be £2.5bn [2]. Whilst sarcopenia was originally diagnosed based on assessment of muscle mass, muscle function measures have been shown to have greater predictive value for clinical outcomes in older adults [3, 4]. As a result, recent guidelines recommend assessment of muscle function as the primary indicator of sarcopenia [5] but a single ‘gold standard’ measure has not been identified. However, assessment of lower limb power using jumping mechanography has been shown to be a more repeatable, sensitive measure with lower practice effects than other measures such as timed up-and-go, chair rises and gait speed [6]. Moreover, the decline in muscle power seems to be (a) more consistent across study populations and measurement methods, and (b) also greater than the decline in muscle size or muscle strength [7, 8].

Physical activity is a key determinant of muscle function across life, such that the pronounced age-related decline in physical activity [9] likely contributes to muscle weakness in older age. Studies of master athletes, who continue to maintain high levels of physical activity in older age [10] allow examination of age-related changes in muscle function in the absence of inactivity. In addition, they permit comparison of the effects of different sporting activities on muscle function. Cross-sectional studies of master athletes suggest that individuals participating in power (*i.e.* sprint or jump) but not endurance-based events have advantages in jump power compared to controls [11, 12]. In addition, these advantages in power athletes were maintained to a similar extent in athletes of both sexes with increasing age [11]. However, to date there have been no longitudinal studies examining muscle function in master athletes. In a recent longitudinal study of bone strength in master athletes, we observed sex and discipline-specific changes in bone outcomes which were not evident in a cross-sectional studies [13]. This highlights the limitations of assessing age-related changes from cross-sectional data.

The aim of this study was to examine longitudinal changes in lower limb muscle function in master power and endurance athletes of both sexes using jumping mechanography. Primarily, we examined lower limb peak muscle power with respect to existing reference data, also published as ‘Esslingen Fitness Index’ (EFI), collected previously by the senior author [14]. We hypothesised that master power athletes of both sexes have advantages in lower limb muscle function relative to normative population values, which are maintained, independent of sex, over time.

## Methods

One hundred and twenty-nine athletes completed muscle function assessments at multiple timepoints. Recruitment was completed in parallel to a longitudinal study of bone strength [13]. Competing athletes were recruited at World, European and British Masters Athletic Championships between 2002 and 2012. Whilst no minimum age-graded performance was selected as an inclusion criterion, recruitment targeted athletes who ranked highest in previous competitions or who had qualified for semi-finals or finals at the current event. Athletes were classified into two different event categories, depending on their self-rated best discipline as power (100/200/400 m, long/high/triple jump, pole vault) or endurance (800 m to marathon) athletes. Self-rated best discipline has previously been shown to be a valid indicator of individual athletic specialisation, showing 95% concordance with age-graded performances [15]. Baseline measures were taken between 2002 and 2009, with follow-up measures taken between 2005 and 2012.

Exclusion criteria were pregnancy and musculoskeletal disorders known to affect the bones, in addition recent injuries which the participant felt could interfere with the test procedure. Participants gave written informed consent before inclusion into the study, which had been approved by the Manchester Metropolitan University Department of Exercise and Sport Sciences Ethics Committee (approval number 2003/12/08). The British, European and World Master Athletics associations have been continuously involved with the design of the study. This was accomplished by discussions with both the associations and the athletes themselves, and by providing feedback and inviting comment on completed studies.

Questionnaires were completed to assess sex, age, self-rated best discipline and self-estimated number of training hours per week, and height and body mass were measured. Athletic performances (best time or jump/vault in the self-selected best event) during each championship were age-graded using the World Masters Athletes age-grading factors and the Age-Graded Performance (AGP) calculator available at http://www.howardgrubb.co.uk/athletics/wmalookup15.html, which expresses performances as a percentage relative to the age-specific world record.

In order to assess lower limb muscle function, participants were asked to complete a series of three separate counter-movement jumps. These tests were performed on a Leonardo force platform (Stratec Medizintechnik GmbH, Pforzheim, Germany) as reported previously [14], with the best jump on the basis of peak power selected. Participants were allowed to use their arms to ascertain balance, but were instructed not to use them to increase jump height. Any trials during which the participant’s hands were elevated above chest height were discarded and the trial repeated. Peak relative (i.e. normalised to body mass) power and force, as well as peak jump height (as determined by the Leonardo software using calculated values of participant’s potential energy) were recorded. In addition, the EFI, which grades the participant’s peak relative power as a percentage compared to age and sex-matched reference data (100% being equivalent to age and sex-matched average values) was also calculated by the software. This reference data is derived from 258 healthy German adults of both sexes using the same experimental procedure [14]. For men, the regression equation for the mean reference peak relative power (in W.kg^−1^) is 77·4–0·62 × age in years, whereas for women it is 55·5–0·42 × age in years. Finally, minimum height (a surrogate for depth of the counter-movement) was recorded in order to detect any difference in jumping strategy between different athletic disciplines or with age.

Statistical analyses were performed using the R statistical environment (version 3.6.2, www.r-project.org). Baseline sex and discipline differences in cohort characteristics and muscle outcomes were compared using one-way ANOVA with Tukey posthoc comparisons. EFI values in each group were compared to normative population values *e.g.* 100 using a one-sample t-test To examine whether muscle function changed with time, linear mixed-effect models were created with a particular muscle outcome as dependent variable, time as fixed effect and participant as random effect, with additional adjustment for training volume, sex and enrolment age. Inclusion of a random participant effect allowed us to account for data clustering caused by differences in number of observations and time between observations. To investigate effects of sex and athletic discipline on changes over time, two and three-way interactions were examined. Interaction terms were removed where *P* > 0.2 on the basis of highest *P*-value until minimal models were obtained, and interactions were identified where *P* < 0.1 for interaction term. Inclusion of quadratic terms was used to test for deviation from linearity for time trends, but there was no evidence of non-linearity. Assuming a medium effect size (partial η^2^ = 0.09) and strong correlation between repeated measures (r = 0.75) given the high precision of jumping mechanography measures (short-term error 3.6% [6]), a sample size of 14 per group would give 80% power to assess within-between factor interactions at an alpha level of 0.05.

## Results

Data from forty athletes were removed from analysis due to missing performance or training data. Therefore, eighty-nine track and field masters athletes (age at baseline 35–90y) were included in this study (Table 1). Their mean age-graded performance was 86 ± 8% (where >80% indicates national-class performance and >90% world-class performance).

**Table 1:** Participant characteristics, separated by discipline and sex presented as mean (SD). AGP – Age-Graded Performance

Variable		Male		Female		Group differences (P)						
		Power (MP)	Endurance (ME)	Power (FP)	Endurance (FE)	Main Effect	Post-hoc Group Comparisons					
At Baseline							FP-FE	ME-FE	MP-FE	ME-FP	MP-FP	MP-ME
n		31	17	25	16							
Age (y)		56.4 (15.4)	66.4 (10.1)	55.8 (12.0)	56 (10.1)	0.036	1.000	0.098	1.000	0.054	1.000	0.048
Height (m)		1.75 (0.07)	1.75 (0.06)	1.65 (0.06)	1.62(0.07)	<0.001	0.453	<0.001	<0.001	<0.001	<0.001	0.991
Body mass (kg)		75.4 (7.5)	68.5 (7.2)	57.5 (5.0)	54.9 (5.6)	<0.001	0.551	<0.001	<0.001	<0.001	<0.001	0.004
Training volume (h/week)		8.0 (3.6)	8.1 (4.4)	7.2 (4.2)	9.3 (8.4)	0.67						
AGP (%)		88.7 (4.9)	80.8 (8.7)	86.4 (7.0)	84.5 (9.1)	0.004	0.827	0.460	0.220	0.067	0.629	0.002
Follow-up												
Time between first and last test (y)		4.3 (2.5)	4.5 (2.1)	5.2 (2.7)	4.2 (2.6)	0.63						
Number of observations	2	27	13	15	11	0.2						
	3	1	2	7	3							
	4	3	2	3	1							
	6	0	0	0	1							

### Cohort Characteristics

Male endurance runners were older than athletes in both power groups (*P* = 0.054 and 0.048 for males and females respectively, Table 1). Men in both groups were taller and heavier than women in both groups (all *P* < 0.001), and male power athletes were heavier than male endurance athletes (*P* = 0.004). There were no group differences in training volume or follow-up time, but the AGP was higher in male power than endurance athletes (*P* = 0.002).

### Baseline Muscle Function Outcomes

Male and female power athletes and female endurance athletes EFI scores were greater (all *P* < 0.01) and male endurance athlete scores lower (*P* = 0.01) than sex and age-matched normative values. Female power athletes had greater EFI than all other groups at baseline (*P* < 0.001, except for male power athletes where *P* = 0.059, Table 2 – individual values for EFI shown in Supplementary Fig. 1). Male power and female endurance athletes had greater EFI than male endurance athletes (both *P* < 0.001). Male power athletes had greater relative jump power than all other groups (all *P* < 0.01), and female power athletes had greater power than male endurance athletes (*P* = 0.023). Male power athletes had greater relative jump force than both endurance groups (*P* < 0.05), and greater jump height than all other groups (*P* < 0.001). There were no group differences in counter-movement depth.

**Table 2:** Jump outcomes at baseline, separated by discipline and sex presented as mean (SD)

Variable	Males		Females		Group differences (P)						
					Main effect	Post-hoc group comparisons					
	Power (MP)	Endurance (ME)	Power (FP)	Endurance (FE)		FP-FE	ME-FE	MP-FE	ME-FP	MP-FP	MP-ME
EFI (%)	123 (17)	91 (13)	134 (19)	112 (15)	<0.001	<0.001	0.003	0.129	<0.001	0.059	<0.001
Relative jump power (W.kg-1)	53.2 (13.0)	34.0 (6.7)	43.0 (9.4)	35.4 (4.9)	<0.001	0.084	0.976	<0.001	0.024	0.001	<0.001
Relative jump force (N.kg-1)	25.6 (3.5)	21.6 (2.8)	23.9 (4.5)	21.9 (2.2)	<0.001	0.273	0.993	0.004	0.148	0.275	0.001
Peak jump height (m)	0.46 (0.12)	0.29 (0.06)	0.35 (0.06)	0.31 (.05)	<0.001	0.377	0.885	<0.001	0.075	<0.001	<0.001
Counter-movement depth (m)	0.26 (0.09)	0.24 (0.09)	0.22 (0.06)	0.21 (.06)	0.070						

*EFI* Esslingen Fitness Index

**Fig. 1:**
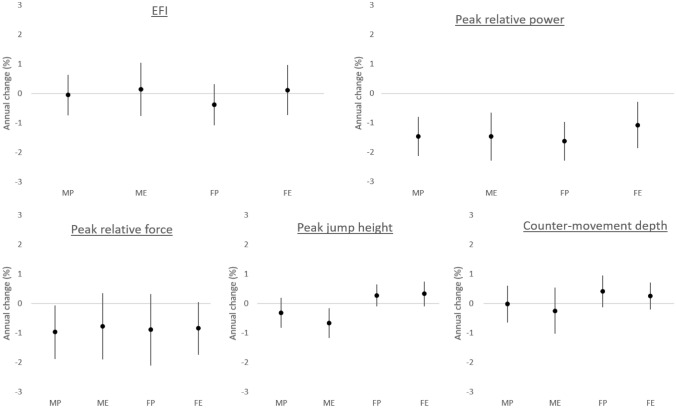
Percentage change per year in jump characteristics (presented as mean ± 95% CI) separated by group, estimated from multiple linear regression analyses adjusted for age at baseline and training volume

In analyses adjusted for age at baseline and training volume, EFI and counter-movement depth were not affected by time, but peak relative power and force and jump height decreased with time (Table 3). Similar results were obtained for unadjusted analyses (not shown), which were also performed with the 40 individuals without complete data (Supplementary Table 1). EFI was lower, and peak relative power, jump height and counter-movement depth higher in males. EFI, relative jump power and force, and jump height were greater in power athletes but there was no association between discipline and counter-movement depth. There was no evidence of time × sex, time × discipline or time × discipline × sex interactions, supported by group-specific estimates of annual change from the same regression models shown in Fig. 1. However, there were sex × discipline interactions for EFI, peak power and peak jump height, such that advantages in power athletes were greater in men than women.

**Table 3:** Results of multiple regression analysis, adjusted for age at baseline and training volume

Variable	Raw values																		
	Main effects												Interactions (*P*-value)						
	Time (year)				Sex (Male)				Discipline (Power)				T*S	T*D	T*S*D	S*D			
	RC	95%CI		P	RC	95%CI		P	RC	95%CI		P				RC	95%CI		P
EFI (%)	−0.22	−0.73	0.29	0.393	−23.0	−33.5	−12.4	<0.001	21.3	13.0	29.6	<0.001	0.545	0.404	0.651	11.60	−0.98	24.18	0.074
Peak relative power (W.kg^−1^)	−0.65	−0.82	−0.47	<0.001	4.2	0.1	8.3	0.049	6.5	3.4	9.6	<0.001	0.644	0.192	0.601	5.99	1.15	10.82	0.017
Peak relative force (N.kg^−1^)	−0.24	−0.37	−0.10	0.001	0.92	−0.21	2.05	0.113	2.79	1.65	3.93	<0.001	0.820	0.324	0.972				0.409
Peak jump height (m)	−0.004	−0.006	−0.001	0.002	0.04	0.01	0.08	0.014	0.05	0.02	0.08	0.002	0.511	0.796	0.515	0.07	0.02	0.11	0.003
Counter-movement depth (m)	0.00	0.00	0.00	0.620	−0.05	−0.07	−0.02	<0.001	0.00	−0.02	0.03	0.711	0.204	0.569	0.773				0.890

*RC* Unstandardized regression coefficient, *CI* Confidence interval, *T* Time, *S* Sex, *D* Discipline, *EFI* Esslingen Fitness Index

## Discussion

The aim of this study was to examine longitudinal changes in muscle function in male and female power and endurance master athletes. We observed that over a mean of 4.5 ± 2.4 years changes in peak power, as well as force and jump height were similar in athletes of both disciplines and sexes. This meant that advantages in peak power relative to normative age and sex-matched values in power athletes and female athletes were maintained over the observation period. In addition, the advantage in peak power and jump height in power compared to endurance athletes was more pronounced in males.

**Fig. 2:**
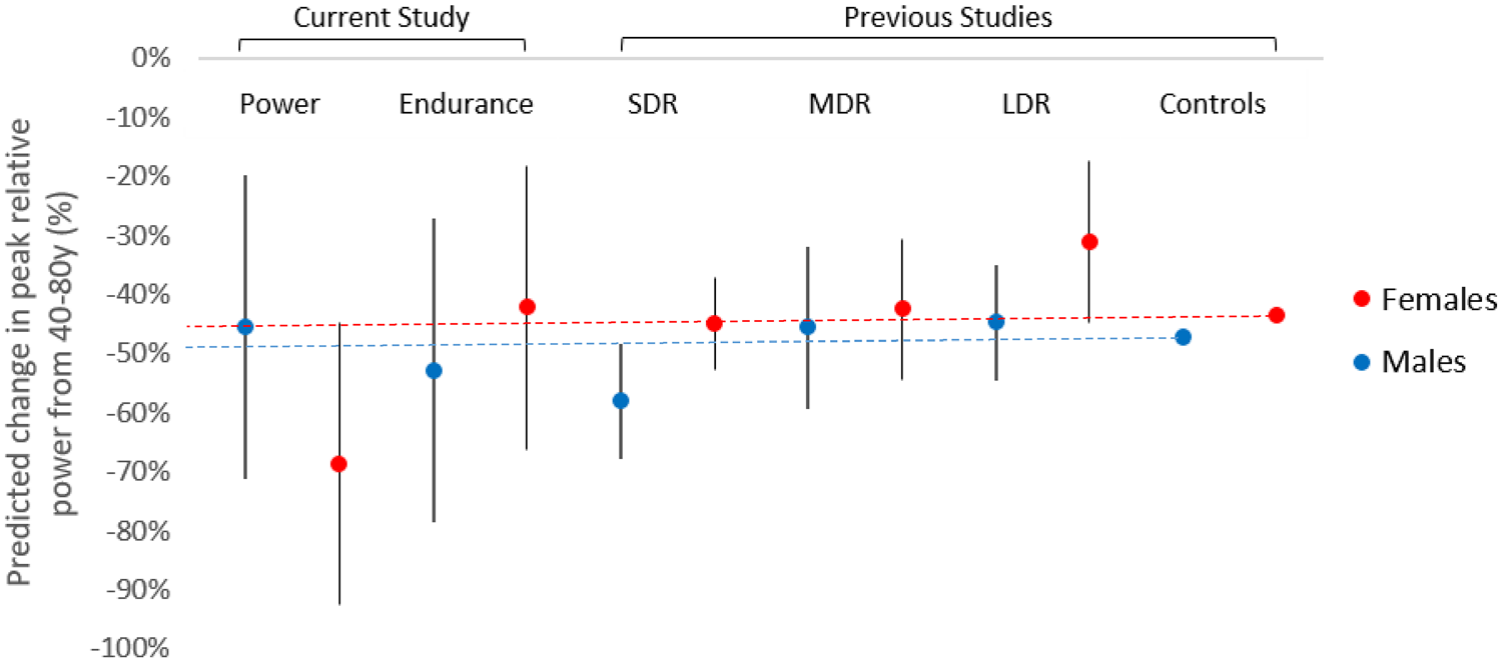
Comparison of findings from the current longitudinal study with those from our previous cross-sectional study in master athletes (*SDR* Sprint runners, *MDR* Middle-distance runners, *LDR* Long distance runners [11], and from our initial study in non-athletic controls [14]. Dashed lines indicate values from control male and female populations

To our knowledge, this is the first longitudinal study of muscle function in master athletes. Comparisons with relative peak power data from cross-sectional studies in runners [11] and non-athletic controls [14] (Fig. 2) suggest that the age-related changes in male athletes in this study were similar to those observed previously. In contrast, age-related decreases in long-distance female runners were less pronounced than those observed in the current study. In addition, power athletes have advantages in peak power over those in endurance disciplines [11, 12]. In contrast, a previous cross-sectional study of master tennis players found that advantages in hand grip in the dominant arm were less pronounced in older players [16]. Whether advantages in peak power relative to age and sex-matched controls differ between sexes had not previously been explored.

It has repeatedly been found that, percentage-wise, running speed declines more rapidly with age for endurance than for sprint running [7]. However, after biomechanical adjustment for kinetic energy, sprint and endurance running power decline with age in a remarkably similar fashion [17]. This is suggestive of a single mechanism for declining endurance and sprint capabilities. A recent cross-sectional study of bio-impedance based assessment of body composition in 256 masters athletes demonstrates that muscle wasting, which goes hand in hand with accumulated adipose tissue offers a viable explanation [18]. With regards to the present study, it is obviously important to consider that the vertical jump test is more related to sprinting capability than to endurance. In this respect, the present findings enhance the view of a general age-related impediment of muscle power across the board of sprint and endurance capabilities. Differences in findings between this study and a previous study in tennis players, where relative advantages in muscle function declined with age relative to the less active control arm [16], may be explained by lower habitual usage of the upper limbs, by occasional use of the non-playing arm in double-handed strokes or the greater power of a within rather than between-individual model. High intensity plyometric training simulating the bouncing movements requiring use of stretch- shortening cycle, fast-twitch muscle fibres and energy storage as in sprint running and jumping have been shown to substantially increase muscle power in older individuals [19].

Another important aspect is that advantages in muscle function in power athletes were greater in male rather than female athletes. In addition greater EFI scores were observed in female power and endurance athletes than their male counterparts, even after adjustment for age and performance. This does not follow results of resistance training studies in older adults in which gains in muscle function were similar between sexes [20]. However, only a small number of plyometric training studies more relevant to power athletic events have been conducted, mainly in younger adults and whilst effects in males were 40% greater than in females the small number of studies meant that there was not strong statistical evidence to support group differences [21]. Given sex differences in use of elastic energy [21] and body composition it is also conceivable that male and female responses to power events and related training may differ. In addition, older women tend to undertake less vigorous physical activity, known to be beneficial for muscle function[22], than older men [23]. Therefore, whilst absolute training volume was similar in athletes of both sexes, this may represent a greater departure from habitual activity levels in female than male athletes.

These results have important clinical significance, given that reduced muscle function, particularly lower limb power in older age is associated with a number of important clinical outcomes [1, 24, 25]. The importance of muscle function is evidenced by greater predictive ability than muscle mass for clinical outcomes in older adults [3, 4], and its inclusion as the primary indicator of sarcopenia in recent guidelines [5]. Consequently, there is a need for a ‘gold standard’ method of muscle function; given that jumping mechanography is a quick, highly-repeatable, sensitive measure of muscle function with lower practice effects than other common clinical tests [6] it would seem to be an ideal candidate. Results of this and previous studies have also shown the ability to apply this technique in long-term longitudinal studies [26]. Moreover, changes in muscle power assessed by jumping mechanography appear to be similar across both cross-sectional and the current longitudinal studies in different populations.

The longitudinal design of this study and recruitment of a reasonably large cohort of elite level master athletes gave us a unique opportunity to examine the effects of aging in the absence of inactivity. Whilst there were baseline group differences in age, which has previously been shown to be linearly associated with muscle function [11, 27], our findings remained robust to additional adjustment. Sprint and power based training has been shown to improve lower limb muscle function in older inactive adults [28], and in master cyclists [29] and runners [30] when added to their usual training schedule. Therefore, whilst there may be a selection bias in the decision to participate as a master athlete, it is highly likely that regular power training contributes to observed group differences. Whilst we used a reference database collected using the same equipment and methodology, the strength of our observations could have been improved by longitudinal collection of data from non-athletes. Use of a German population reference database may limit the generalisability of our results to athletes from other countries. However, given that the focus of our study was age-related changes, we are reassured by the almost identical age-related decline in jump power identified in a large population of Japanese adults using the same assessment [31]. Whilst the mean, variance and age range were similar between the current longitudinal study and our previous cross-sectional study, we acknowledge that caution must be exercise when comparing the two types of study.

In elite master athletes, similar longitudinal changes in muscle function as assessed by jumping mechanography were observed in male and female power and endurance athletes. This is in contrast to findings of a similar study of bone strength in members of this cohort, where we observed greater maintenance of bone mineral content in male than female athletes and in power than endurance athletes [13]. Given baseline differences, this meant that power athletes maintained an advantage in lower limb peak power relative to normative reference values. The results of this study suggest that advantages in master power athletes in particular with respect to age and sex-match normative values, which includes the contribution of power training, are maintained over time. Future studies should examine whether gains in lower limb function in less active individuals are maintained with prolonged training, as indicated by the results of the current study and previous cross-sectional observations [11].

## Supplementary Information

Below is the link to the electronic supplementary material.

Supplementary file1 (DOCX 66 kb)

## Data Availability

Data are available to bona fide researchers upon request to the corresponding author.
