# Supplementary material for: Age-Related Declines in Lower Limb Muscle Function are Similar in Power and Endurance Athletes of Both Sexes: A Longitudinal Study of Master Athletes
Source: Calcif Tissue Int. 2021 Sep 9;110(2):196–203. doi: 10.1007/s00223-021-00907-3 (PMC8784358; doi:10.1007/s00223-021-00907-3)
Supplement: Supplementary file 1 — Supplementary file1 (DOCX 66 kb) [file 223_2021_907_MOESM1_ESM.docx]

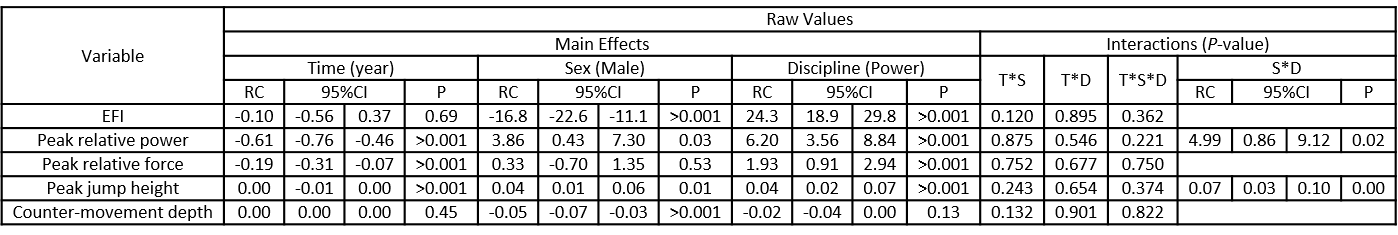

Supplementary Table 1. Results of multiple regression analysis in 129 individuals (47 MP, 23 ME, 30 FP, 29 FE), consisting of 89 individuals from the main analysis with complete data, and 40 individuals with incomplete training or Age-Graded Performance (AGP) data. RC – unstandardized regression coefficient, CI – confidence interval, T – Time, S – Sex, D – Discipline, EFI – Esslingen Fitness Index.


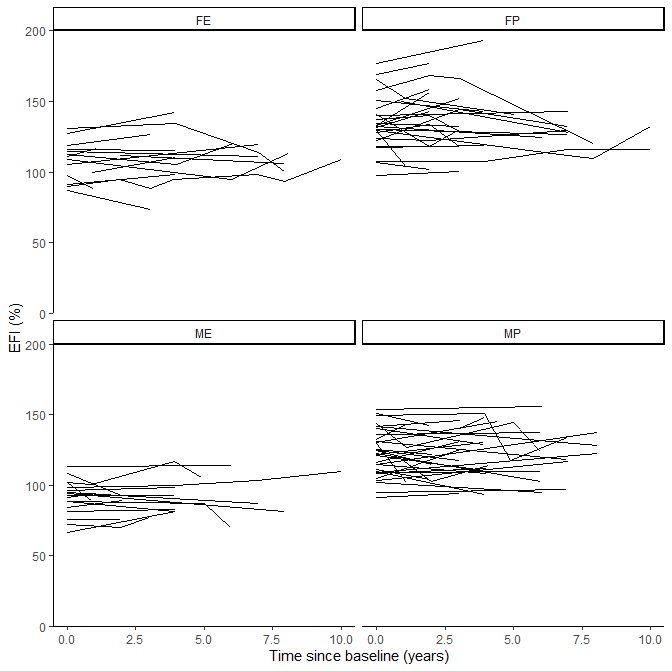


Supplementary Figure 1. Individual values for Esslingen Fitness Index (EFI) throughout data collection period, separated by sex and discipline
